# The Molecular and Cellular Basis of Hutchinson–Gilford Progeria Syndrome and Potential Treatments

**DOI:** 10.3390/genes14030602

**Published:** 2023-02-27

**Authors:** Noelle J. Batista, Sanket G. Desai, Alexis M. Perez, Alexa Finkelstein, Rachel Radigan, Manrose Singh, Aaron Landman, Brian Drittel, Daniella Abramov, Mina Ahsan, Samantha Cornwell, Dong Zhang

**Affiliations:** Department of Biomedical Sciences, College of Osteopathic Medicine, New York Institute of Technology, Old Westbury, NY 11568, USA

**Keywords:** Hutchinson–Gilford progeria syndrome, HGPS, progeria, aging, laminopathy

## Abstract

Hutchinson–Gilford progeria syndrome (HGPS) is a rare, autosomal-dominant, and fatal premature aging syndrome. HGPS is most often derived from a de novo point mutation in the *LMNA* gene, which results in an alternative splicing defect and the generation of the mutant protein, progerin. Progerin behaves in a dominant-negative fashion, leading to a variety of cellular and molecular changes, including nuclear abnormalities, defective DNA damage response (DDR) and DNA repair, and accelerated telomere attrition. Intriguingly, many of the manifestations of the HGPS cells are shared with normal aging cells. However, at a clinical level, HGPS does not fully match normal aging because of the accelerated nature of the phenotypes and its primary effects on connective tissues. Furthermore, the epigenetic changes in HGPS patients are of great interest and may play a crucial role in the pathogenesis of HGPS. Finally, various treatments for the HGPS patients have been developed in recent years with important effects at a cellular level, which translate to symptomatic improvement and increased lifespan.

## 1. Introduction 

Normal aging refers to a time-dependent deterioration of cells, tissues, and overall physiological functions accompanied by an increased risk of various pathologies, including cancer, cardiovascular disease, diabetes, and neurodegeneration [1]. Pathological aging, most notably the progeroid syndromes, can rapidly accelerate these risks [1,2]. Attempts to examine their causes have revealed major gaps in our current understanding of how the aging mechanism works. Furthering our knowledge of the underlying molecular and cellular processes of abnormal aging is necessary to gain insight into the development and detection of aging-related diseases, to identify novel therapeutic approaches, and overall to improve human health during aging [1].

Progeroid syndromes, also referred to as premature aging disorders, encompass a heterogeneous group of rare, highly fatal, and hereditary diseases that appear to recapitulate multiple phenotypes of advanced physiological aging extremely early on in development [3]. One of the earliest reported cases of a progeroid syndrome was first published in 1886 and depicted some of the harsh clinical manifestations of the disease in a three-year-old child [4]. This report described what is now referred to as Hutchinson–Gilford Progeria Syndrome (HGPS). Investigations of the molecular basis of these progeroid diseases have revealed perturbations of critical cellular processes such as DNA replication, DNA repair, and the formation of nuclear membrane architecture [5]. Interestingly, new evidence suggests that the physical properties and connections at the nuclear–cytoskeletal interface directly contribute to numerous cellular functions, including mechanotransduction [6,7]. Mechanotransduction is the process by which cells convert mechanical signals from the extracellular matrix into biochemical signaling pathways transmitted from the cytoskeleton to the nucleus and across the nuclear envelope to the nuclear lamina and chromatin. Notably, this has been shown to result in downstream signaling and altered gene expression [6,7]. HGPS, in particular, was found to have its roots in a unique de novo heterozygous silent mutation in the human nuclear lamin A/C (*LMNA*) gene, resulting in a 1824C>T single base substitution at the *LMNA* codon 608, which led to the accumulation of a truncated protein referred to as ‘progerin’ [8]. Interestingly, lamins have been shown to play a central role in initiating mechanotransduction signaling [6,7]. Thus, research into progeroid syndromes such as HGPS provides a unique opportunity to characterize cellular mechanisms that contribute to normal aging and nuclear morphology while also exploring how alterations in mechanotransduction pathways contribute to aberrant downstream signaling and altered gene expression [2,5]. Additionally, because premature aging syndromes are unique model systems that can be used to facilitate the study of normal aging, their study can potentially further our understanding of age-associated diseases [2].

In this review, we initially focus on our understanding of the molecular basis of HGPS with an added emphasis on uncovering the newly emergent roles of different types of epigenetic changes and whole-genome alterations. The importance of examining epigenetic alterations on manifestations of HGPS is particularly critical for two primary reasons: (1) understanding the connection between normal and abnormal aging; and (2) potentially revealing novel therapeutic avenues. Specifically, we limit our scope to the patterns of DNA methylation events, histone modifications, and lamin-associated domains (LADs). Lastly, we summarize the current state of therapeutic options for HGPS patients and possible directions for future research. 

## 2. Molecular Pathogenesis of HGPS

### 2.1. Introduction to Lamins and Laminopathies

**Figure 1:**
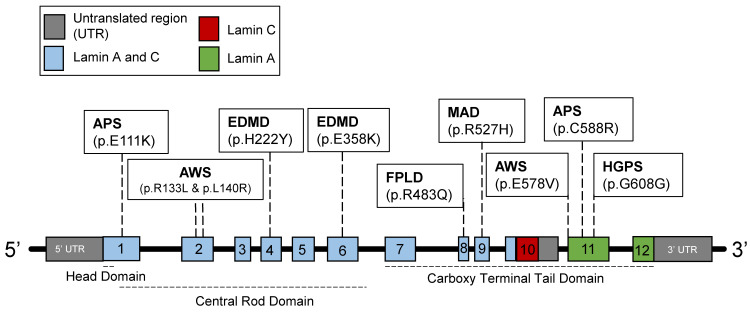
The structure of the human *LMNA* gene. Lamin C is encoded by exons 1 to 10, while lamin A is encoded by exons 1 to 12. Various mutations identified correlate to pathologies that cause progeroid syndromes and/or segmental diseases affecting connective tissues. EDMD, Emery–Dreifuss muscular dystrophy. APS, atypical progeroid syndromes. HGPS, Hutchinson–Gilford progeria syndrome. AWS, atypical Werner syndrome. FPLD, Dunnigan familial partial lipodystrophy. MAD, mandibuloacral dysplasia.

To date, roughly 500 disease-causing mutations have been identified in the human *LMNA* gene, with each mutation resulting in unique cell- and tissue-specific manifestations [9] (Figure 1). Nevertheless, a common theme amongst these laminopathies is that they disrupt pathways in the normal development of connective or mesenchymal-derived tissue, which includes skeletal muscle, vascular smooth muscle, and bone [10,11]. Furthermore, some *LMNA*-mutated conditions only cause tissue-specific dysfunction, which include mandibuloacral dysplasia, familial partial lipodystrophy II, and Emery–Dreifuss muscular dystrophy [12,13,14,15,16] (Figure 1). The others cause premature aging syndromes, such as atypical Werner syndrome (adult progeria), as well as other atypical progeroid syndromes [17,18,19] (Figure 1). HGPS is one of the most widely studied laminopathies, likely due to its resemblance to physiological aging. Notably, cellular aging defects attributed to the effects of progerin on the nuclear lamina observed in HGPS overlap significantly with those observed in normal aging [20]. In fact, defects in the nuclear lamina resulting from progerin accumulation have been directly linked to the twelve hallmarks of aging, including genomic instability, telomere attrition, epigenetic alterations, loss of proteostasis, disabled macroautophagy, deregulated nutrient sensing, mitochondrial dysfunction, cellular senescence, stem cell exhaustion, altered intercellular communication, chronic inflammation, and dysbiosis [1]. 

### 2.2. Physiological Functions of the Lamins

**Figure 2:**
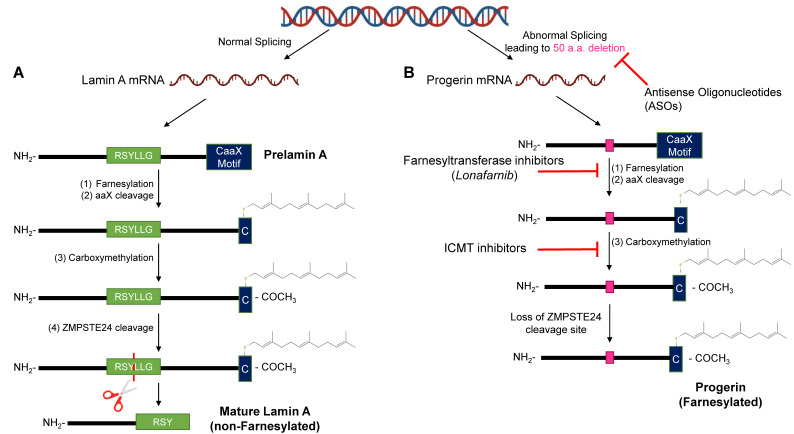
The differences between processing/modifying a normal prelamin A protein and the progerin mutant. (**A**): Normal splicing generates a prelamin A protein, which has a terminal CaaX box. The “C” denotes cysteine, “a” is an aliphatic residue, and “X” is any amino acid. The cysteine residue is initially prenylated by a 15-carbon isoprenoid unit. Subsequently, the aaX motif is cleaved by ZMPSTE24, and then a carboxyl-methyl group is added by isoprenylcysteine carboxyl-methyltransferase (ICMT). The final cleavage step is performed by ZMPSTE24, removing 15 amino acids from the C-terminal region including the farnesylated cysteine. (**B**): 1824 (C>T) mutation at the human *LMNA* gene generates a cryptic donor splice site. The cryptic donor splice site is improperly recognized by the spliceosome, leading to a 50 amino acid (a.a.) deletion in the prelamin A protein including the ZMPSTE24 cleavage site (pink rectangle). The next few steps mirror normal lamin A production. The final ZMPSTE24-mediated step does not occur due to absence of the ZMPSTE24 cleavage site, resulting in a permanently farnesylated and carboxyl-methylated molecule.

As mentioned above, HGPS is most commonly due to a de novo mutation in the human *LMNA* gene [8]. The nuclear side of the inner nuclear membrane is lined by a ~15 nm structure referred to as the nuclear lamina [21]. The principal components of the nuclear lamina are type V intermediate filaments, consisting of a family of proteins termed lamins, which are all encoded by the *LMNA* gene [22]. The *LMNA* gene initially generates prelamin A, which is post-translationally modified to become lamin A [23] (Figure 2A). The post-translational modifications include (1) farnesylation at the cysteine residue at the carboxyl terminal CaaX motif; (2) cleavage of three amino acids (aaX) at the C-terminus by ZMPSTE24; (3) addition of carboxyl-methyl group to the farnesylated cysteine; and (4) the final cleavage of 14 amino acids upstream of the farnesylated cysteine by ZMPSTE24 [24,25,26,27,28,29] (Figure 2A). Lamin C is generated directly from the mRNA and does not undergo any post-translational processing [30]. The particular lamin A/C subtypes play multiple different roles in normal cell processes and have a pathological role when these normal processes become disrupted in HGPS [31].

**Figure 3:**
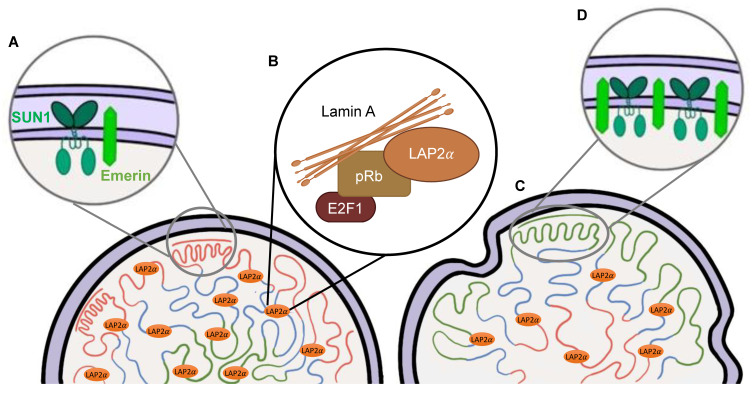
Nuclear structure, nuclear membrane protein, and chromatin alterations with the expression of progerin. (**A**): Emerin and SUN1 are inner nuclear membrane proteins that interact with lamin A. (**B**): LAP2α is a protein localized to the nucleoplasm also in association with lamin A. Furthermore, they both play an important role in regulating the phosphorylated retinoblastoma (Rb) protein (pRb) and E2F1 (a transcription factor). (**C**): After the expression of progerin, chromatin alterations are identified with the typical heterochromatin localizing inside the nucleoplasm. In addition, there are nuclear shape aberrations as well as decreased LAP2α expression. (**D**): Increased SUN1 and emerin expression correlated with progerin presence at the nuclear membrane.

Early studies suggested that lamins were simply structural components of the nuclear envelope; however, more recent studies have begun to uncover the lamins’ complex role as key regulators of a variety of cellular processes, notably through their role in mechanotransduction pathways [32,33,34,35]. Due to their unique structure and location at the nuclear periphery, lamins can directly and indirectly interact with numerous nuclear membrane proteins, termed lamin-associated proteins (LAPs) [36,37] (Figure 3A). Furthermore, LAP2α is an LAP that is primarily located in the nucleoplasm [38] (Figure 3B). Additional proteins that lamins interact with include transcription factors, chromatin, chromatin regulators, signaling molecules, and splicing factors [39,40,41,42,43] (Figure 3B). These proteins form a complex lamin meshwork that is involved in various cellular functions [32,39,43,44,45]. Currently, lamins have been shown to play a role in maintaining nuclear integrity, regulating transcription through their interactions with transcription factors and signaling transduction pathways, and contributing to the 3D genome organization through the scaffolding of lamin-associated domains (LADs) [32,39,43,45] (Figure 3B). This highlights the interconnected role of chromatin and lamins with various mechanical properties at the nucleus.

### 2.3. Disruptions of the Functions of Lamin A in HGPS

In HGPS, the point mutation 1824 (C>T) is a silent base substitution (Gly608Gly or G608G). However, it produces an active cryptic splice donor site [8] (Figure 1 and Figure 2B). When the spliceosome recognizes this cryptic splice donor site, it removes 50 amino acids at exon 11 from lamin A [8] (Figure 2B). The initial steps (1) to (3) of the post-translational modifications are the same for the mutated prelamin A [46] (Figure 2B). However, the final cleavage step does not occur due to the absence of the ZMPSTE24-endoproteolytic site in the missing 50 amino acids; thus, progerin is generated instead of the mature lamin A [46] (Figure 2B). The progerin is permanently farnesylated and is anchored to the nuclear membrane [47]. Consequently, progerin induces nuclear abnormalities, such as lobulation [47] (Figure 3C). Other cellular alterations are also observed, including telomere shortening, defects in DNA repair, dysregulated gene expression, genomic instability, and premature senescence [31,48,49].

## 3. Progerin Expression Phenotypes

### 3.1. Nuclear Abnormalities

Nuclear abnormalities are commonly observed in normal aged cells and in HGPS cells. These aberrations are mainly characterized by a loss of shape with blebbing, invaginations, and a possible nuclear membrane rupture [50,51,52,53,54,55,56] (Figure 3C). In HGPS, nuclear lamina defects manifest due to the accumulation of farnesylated progerin [47]. An additional protein player involved is SUN1 (one of the LAPs), which is required for progerin-induced nuclear defects [57] (Figure 3D). Other factors include ESCRT-III, an integral endosomal sorting complex involved in repairing damaged NMs [58]. This highlights the complex nature of nuclear shape maintenance in HGPS cells [58]. Similar to normal aged cells, progerin is also sporadically expressed and is responsible for their nuclear abnormalities [56]. As a result of these defects, the nuclei become stiffened and are unable to respond normally to mechanical stress [59]. This manifests as increased nuclear membrane ruptures in vascular smooth muscle cells (SMCs) [53]. This could explain why tissues like skin, cardiac muscle, and the vasculature are largely affected, due to their intense mechanical stress, while neurological tissues are not [60,61]. Conversely, a study using an HGPS fish model established that longevity and nuclear abnormalities do not influence each other; although it is important to note that the fish model did not fully recapitulate the wild-type phenotype [62]. However, the reversal of these nuclear abnormalities has been shown to improve clinical phenotype and longevity in mouse models [63]. The widespread deleterious effects observed with increased nuclear alterations can be due to the nuclear lamina’s importance in anchoring and organizing the genome to regulate gene expression [40,64]. Interestingly, the rupture of the lamina was also shown to influence the localization of proteins involved in DNA repair [65]. Mislocalization of these proteins caused an increase in unresolved DNA lesions, and this is consistent with DNA damage levels in instances of increased cellular senescence as well as normal aging [66,67]. 

### 3.2. Defects in DNA Damage Response

**Figure 4:**
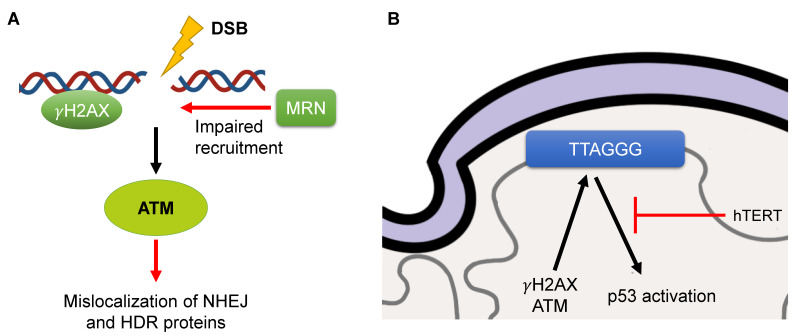
Aberrant DNA damage response (DDR) and telomere positioning in progerin-positive cells. (**A**): Initial and downstream DDR signaling perturbed in HGPS cells. (**B**): Decreased mobility of telomeres identified, with more than half in proximity of the nuclear lamina. DDR and p53 activation is up-regulated at telomeres. p53 is responsible for much of the senescent phenotype of HGPS cells, and its activation is abrogated by the expression of hTERT.

HGPS fibroblasts have been consistently observed to have increased DNA damage response (DDR) markers (53BP1 and γH2AX foci) [68,69] (Figure 4A). Therefore, the DDR is persistently activated, with increased levels of phosphorylated Chk1 and Chk2 (pChk1 and pChk2), two important downstream DDR kinases, as well as ATM and ATR, two important upstream DDR kinases [49] (Figure 4A). The activation of these DDR kinases induces a senescent state [49]. ATR is also shown to be mislocalized in HGPS cells [70]. In addition, components of the MRN complex (Rad50, NBS1, and MRE11), a key activator of ATM, are impaired in their recruitment to double-strand breaks (DSBs) [69] (Figure 4A). Furthermore, KAP-1, a downstream target of ATM involved in the recruitment of various DNA repair proteins, was altered in ZMPSTE24-deficient MEFs, and knockdown of KAP-1 resulted in improved DNA repair [71]. Interestingly, the progerin-generated DSBs were not resolved by endogenous DNA repair factors, but those created by chemotherapy agents were. Progerin also disrupts later steps of the DDR, with alterations in canonical nonhomologous end-joining (cNHEJ) and homology-dependent repair (HDR) (Figure 4A). Rad51, a key player in HDR, did not colocalize with γH2AX foci in HGPS cells [69] (Figure 4A). The DNA-PKcs holoenzyme, a key player in cNHEJ, was also shown to have decreased expression in HGPS SMCs [72] (Figure 4A). 

On the other hand, one group showed that progerin abolished PARP1 expression and led to an induction of cNHEJ activity [73] (Figure 4A). One key reason that progerin is likely able to influence the DDR pathway in such a multifactorial manner is due to the XPA protein, a key player in nucleotide excision repair (NER), being in close proximity to DSBs. After the knockdown of XPA, there were fewer DSBs, as well as increased recruitment of Rad51 and Ku70 (the latter is another key player in cNHEJ) to those sites in HGPS cells [69]. Furthermore, PCNA was also mislocalized to the nuclear membrane with progerin, potentially another cause of DNA damage [74]. One study indicated that improvements in nuclear shape from farnesyltransferase inhibitor (FTI) treatment did not result in a concomitant decrease in DSBs and impairment of repair factors [49]. However, another study did observe this outcome, but only for progerin-expressing cells that received ionizing radiation [75]. The interrelation of structure and DNA damage is an interesting phenomenon and should be further explored.

In normal aging, DNA damage is a prominently observed phenotype due to both exogenous and endogenous sources of damage [66,67,76]. Additionally, aged cells have a reduced capacity to repair DNA lesions, with many DNA repair pathways being impaired, including NER and base excision repair (BER) [77,78]. Further parallels between premature aging in HGPS and normal aging include dysfunction at telomeres as well as premature senescence [79,80]. DDR causes telomeres to express telomeric non-coding RNAs (tncRNA), contributing to the detrimental phenotype; inhibition of the tncRNAs improved cellular manifestations and lifespan in a transgenic HGPS mouse model [81].

### 3.3. Telomere Attrition

DDR and telomere attrition are intrinsically linked [82]. Telomere shortening and subsequent senescence has been consistently observed across several cell and tissue types during normal aging [83]. Two mechanisms have been identified to induce senescence from telomere alterations. The first is when telomeres become uncapped, due to the lack of a functional Shelterin complex, resulting in the activation of DDR and senescence [84]. The second is that the shortened telomeres lead to the activation of the p53 pathway and inhibition of normal cell-cycle progression [85]. Of note, DNA damage has also been shown to be associated with aged, but not necessarily shortened, telomeres [82,86]. Telomeres have been demonstrated to be altered with progerin expression, with generally reduced lengths [48]. Additionally, the importance of telomeres in progerin’s phenotypic expression has been studied. One group used telomerase (hTERT)-expressing HGPS cells and demonstrated a significant lack of β-galactosidase staining (a known cellular senescence marker), along with prominent down-regulation of the p53 pathway [87,88] (Figure 4B). Another study identified that improvement in the senescent phenotype only occurred in late-passaged HGPS cells [89]. In addition, much of progerin-induced damage to the telomeres was alleviated by the expression of hTERT, which abrogated γH2AX foci and ATM signaling [87] (Figure 4B). There were also whole-chromosomal effects in HGPS cells, with telomeric fusions and loss as well as the formation of chromatin bridges [87]. 

The overall relationship between the progerin phenotype and the effect on telomere characteristics still requires further explanation. One group observed that progerin-induced DNA damage was localized primarily to sites of collapsed replication forks as opposed to telomeres [74]. Furthermore, they identified that the premature senescence component had been shown not to be associated with telomere shortening and was instead due to the activation of p53, as the main cause of replicative arrest, through a mechanism that is currently unknown [74]. Other interesting manifestations include hTERT suppressing progerin expression in HGPS cells [90]. These different outcomes regarding the importance of telomeric attrition in the HGPS phenotype warrant further investigation. Additionally, the connection between telomeres and the lamina cannot be ignored, as telomeric mobility is decreased due to increased progerin anchorage at the nuclear membrane [91] (Figure 4B). In addition, LAP2α has been identified to have decreased association with telomeres [88]. As mentioned previously, in aged individuals, normal cells can begin to express progerin. Telomere shortening and concomitant senescence has been identified as causes for the aberrant splicing in normal fibroblasts to produce progerin [90]. Overall, there is conflicting evidence regarding the importance of telomere attrition in HGPS, but it has been identified as a major feature in both HGPS and aged cells.

## 4. Tissue/Organ Dysfunction

As stated, HGPS can serve as a model for aging, since many aspects of the clinical manifestations are shared. However, some important differences do exist. One notable difference is that HGPS is a segmental disease that primarily afflicts mesenchymal-derived tissues such as bone, vascular smooth muscle, and skeletal muscle [10,11]. Another interesting observation is that progeria patients do not typically develop cancer, possibly due to a potentially protective mechanism of bromodomain-containing protein 4 (BRD4) [92]. Below is a detailed account of the various symptoms of HGPS. 

Patients with HGPS typically appear normal at birth [93]. By the age of one or two, severe growth retardation along with many other abnormal physical features begin to manifest [93]. Generally, HGPS patients initially present with a failure to thrive. Mean weight, height, and head circumference have been documented to be below the third percentile for HGPS children along with decreased subcutaneous fat [94]. 

Regarding cardiovascular manifestations, HGPS patients succumb to stroke or MI due to atherosclerosis at a median age of 14.6 years [95]. Cerebral angiography reveals severe stenosis of the middle cerebral, vertebral, and basilar arteries [94]. Transient ischemic attacks have been shown to be common among these patients [94]. At a tissue level, there is marked adventitial fibrosis in arteries and veins along with a dense rim of collagen [96]. The most evident adventitial changes are typically seen in the aorta and the coronary arteries. Nonspecific inflammation has also been seen in the adventitial perivascular fibrosis. Similar findings are present around non-cardiac vessels, including arteries of the spleen, salivary glands, lymph nodes, lymphatic vessels, and pulmonary arteries. The central veins of the liver and the portal triad, epicardial, and hilar lymph node veins have also exhibited extensive perivascular tissue fibrosis [96]. These abnormalities can lead to abnormal vital signs, such as increased systolic and diastolic blood pressure, increased heart rate, and QT prolongation on electrocardiogram [94]. 

Craniofacial abnormalities are very prominent in HGPS patients, due to the dysfunction of bones of the cranium. Specifically, abnormalities involving the calvaria, skull base, and soft tissue of the face and orbit have been observed [97]. Oral abnormalities included hypodontia, ankyloglossia, arched palate, double rows of teeth, and delayed tooth eruption [94]. Furthermore, secondary incisors can be located lingually and palatally in the mandible and maxilla, respectively, rather than erupting in place of the primary incisors, as seen in normal development. There was also delayed tooth eruption seen in both primary and secondary teeth [94]. Progressive mandibular maldevelopment was also seen in patients with HGPS [98]. Thus, a wide spectrum of alterations is visualized in the cranium. 

Bony abnormalities are also identified in the rest of the skeleton [94]. This includes decreased bone mineral density, specifically of the lumbar spine, as well as clavicular resorption [94,98]. Thinning and tapering of ribs is observed as well, resulting in an apex narrowing and a pyramidal configuration of the thorax. Furthermore, acroosteolysis (osteolysis of distal phalanges) has been identified, which severely progresses with increasing age [98]. Patients also present with joint contractures, specifically abnormal joint extension contractures and contractures of the knee or ankle [94,98]. Abnormal range of motion is another classic presentation of HGPS patients, typically in at least three peripheral joints [94]. Reduced range of motion was seen in the wrist, ankle, hip, and spine. Radiologic examinations showed distal joint abnormalities, such as coxa valga [94]. Despite all these abnormalities, normal phenotypic musculoskeletal findings are observed, including normal bone age, growth plates, joint space width, and muscle volume remaining proportional to body mass [94,98]. Additionally, it was found that HGPS patients do not exhibit osteo- or rheumatoid arthritis, periarticular erosions, nor proliferative changes such as osteophyte formation [98].

Although HGPS is a segmental disease, it can cause disease in non-mesenchymal-derived tissues [10,11]. Ophthalmologic findings in HGPS patients include hyperopia, corneal dryness, dry-eye syndrome, and keratopathy, while intraocular pressures are normal. Regarding skin abnormalities, HGPS patients tend to have sclerotic skin, dimpling and mottling of the skin, circumoral cyanosis, and fingertip tufting, as well as prominent cutaneous vasculature such as prominent scalp veins. Alopecia is another common presentation. Speech and language are also impaired with labial weakness as well as diminished lingual range of motion and strength. Furthermore, conductive hearing loss was identified in most patients [94]. 

HGPS clinical manifestations are drawn from the various defective molecular processes and alterations mentioned prior. However, they fail to tell the whole story. Recently, the epigenetic landscape has been investigated in many diseases as a source of pathogenesis [99,100]. Specifically, changes at the nuclear lamina drive alterations in gene expression through lamin-associated domains (LADs) [101]. This could serve as another piece to the complex interplay of progerin at the cellular level. 

## 5. Epigenetic Alterations in HGPS

### 5.1. Histone Modifications 

Studies have revealed that normal aging and HGPS are associated with severe epigenetic aberrations including histone modifications, histone variants, DNA methylation, chromatin remodelers, and chromatin architectures [1,54,72,101,102]. However, the complex interplay between these factors and the underlying definitive molecular mechanisms remains largely unclear. 

One of the most widely recognized epigenetic changes observed in both normal aging and premature aging syndromes is profound alterations in heterochromatin markers [102,103]. Specifically, fibroblasts from patients with HGPS are known to exhibit a loss of transcriptionally repressed peripheral heterochromatin [102]. This is evidenced by a reduction of histone H3 trimethylation at lysines-9 and -27 (H3K9me3 and H3K27me3) as well as a down-regulation of H3K27me3 methyltransferase, EZH2, and associated protein heterochromatin protein 1 (HP1) [20,56,101,102,104]. These histone markers are typically distributed characteristically throughout the genome, with H3K27me3 noted to be associated with the inactivated X chromosome in females with HGPS [105]. It is well-established that these changes contribute to heterochromatin dissociation from the nuclear lamina, which ultimately results in the disruption of spatial nuclear compartmentalization [106,107]. Hi-C experiments confirmed that the down-regulation of histone markers contributes to the disorganization of active and inactive chromatin domains in late passage HGPS cells [101,106,108]. Conversely, the heterochromatin marker H4K20me3 is upregulated in HGPS cells [101,102,109]. Increased H4K20me3 blocks telomere elongation, which supports the observed telomeric dysfunction and subsequent accelerated senescence observed in HGPS.

The loss of heterochromatin has also been shown to coincide with the down-regulation of many proteins that contribute to epigenetic silencing, including EZH2, PRC2 (a member of the polycomb recessive complex), HP1⍺, and SUV39H1 [54,55,56,102,106]. Interestingly, in a yeast two-hybrid screening using the lamin A/C-terminal region as the bait, which overlaps the deleted 50 amino acids in progerin, two evolutionarily conserved histone binding proteins (RBBP4 and RBBP7) were found to interact with lamin A [54]. Notably, RBBP4 and RBBP7 are shared subunits of several multi-protein complexes including the nucleosome remodeling and deacetylase (NURD) complex and the polycomb PRC2 complex, which are involved in establishing heterochromatin and are down-regulated in HGPS cells. Specifically, the NURD chromatin remodeling complex has been identified as a significant modulator of aging-associated chromatin defects in both premature and normal aging. Knockdown of individual NURD subunits using siRNA resulted in the reduction in H3K9me3 and increased markers of DDR, recapitulating chromatin defects associated with aging [54]. Furthermore, the reduction of HDAC1 protein in HGPS cells also suggests a loss of HDAC1 deacetylase activity [110,111]. Treatment with histone deacetylase inhibitors (HDACi) resulted in decompaction of heterochromatin and subsequent DNA damage and senescence [110,111]. 

The exact mechanism of how progerin accumulation causes the drastic chromatin alteration remains largely unclear. Thus far, it has been established that progerin-induced heterochromatin loss is not a result of cell senescence [88,102]. A doxycycline-inducible system was developed to express progerin in isogenic primary and hTERT-positive human dermal fibroblasts. While hTERT prevented progerin-induced premature senescence, the expression of progerin in telomerase-positive immortalized cells did not prevent heterochromatin loss and nuclear abnormalities [88,102]. Moreover, expressing progerin at different cell cycle stages revealed that progerin triggers heterochromatin decompaction in growth-arrested G0 cells in stark contrast to DNA damage, which accumulates exclusively during DNA replication. Further evidence was seen by inducing the expression of progerin throughout different cell cycle stages, such as G1-arrested cells [112]. This study determined that progerin expression and subsequent heterochromatin loss is independent of DNA replication and mitosis. Progerin-induced DNA damage occurred preferentially in cells with low levels of heterochromatin and exclusively during late stages of DNA replication, prior to chromosome condensation [112]. Visualization of progerin levels, heterochromatin levels, and DNA damage at single-cell resolution also revealed that low levels of heterochromatin are more prone to DNA damage, while progerin abrogation in G0 cells restored heterochromatin levels and prevented accumulation of DNA damage [55,112]. Overall, while histone modification is generally a hallmark of progerin-positive cells, it is not always connected with other aspects of progerin-induced cellular damage.

### 5.2. DNA Methylation

Aberrant DNA methylation has also been observed in HGPS. A survey of genome-wide CpG methylation in HGPS fibroblasts identified 586 differentially methylated autosomal genes in HGPS fibroblasts compared to the control fibroblasts [72]. Interestingly, when using DAVID analysis (an integrative analysis of large gene lists), these genes were enriched for twenty-one gene ontology terms, mostly relating to development and transcriptional regulation [72,88,101,102,106]. Surprisingly, when comparing methylation differences between HGPS-induced pluripotent stem cells (HGPS-iPSCs) that did not express progerin with the control iPSCs, only 33 differently methylated autosomal genes were found, with no significant functional enrichment observed [72]. Thus, the presence of progerin in HGPS fibroblasts may induce methylation changes, whereas the down-regulation of progerin in HGPS-iPSCs appears to ameliorate the epigenetic changes in DNA methylation. Another study identified significant DNA methylation changes in age-related genes, but these patients had non-classical progeroid laminopathies [101,113]. Recently, it has been shown that some HGPS fibroblasts display an increased “DNA methylation age.” The group used the methylation status of 391 genomic loci to estimate the biological age, which may suggest a significant degree of underlying methylation changes that are in need of elucidation [114]. Importantly, altered DNA methylation patterns have been widely observed as biomarkers of physiologic aging as well. In fact, loss of DNA methylation in lamina-associated, late-replicating regions, referred to as partially methylated domains (PMDs), has recently been identified as a pan-tissue biomarker of cellular aging and thus may be an important area to explore in premature-aging conditions [101,115]. 

### 5.3. LADs As a Potential Link 

Another recent area of interest is lamin-associated domains (LADs), which are heterochromatic regions of the DNA in close contact with the nuclear lamina (Figure 3B). It is widely recognized that in HGPS cells, the accumulation of progerin distorts the nuclear lamina, resulting in severe changes in nuclear organization and nuclear compartmentalization (Figure 3C) [47]. Additionally, the spatial compartmentalization of active and inactive chromatin domains is disrupted in late-passage HGPS cells [101,106,108] (Figure 3C). Furthermore, using SAMMY-Seq, one group was able to observe disturbed chromatin organization, even before observed senescent markers and alterations in nuclear shape [116]. 

LADs are a potential avenue to explain the epigenetic changes involved in both physiological and pathological aging. They have been shown to contribute to the spatial organization of the genome, specifically playing active as well as passive roles in nuclear lamina tethering, transcription regulation, and genome replication [117] (Figure 3C). LAD patterns appear to be partially conserved between cell types across species; however, they also display cell-specific variations based on cell type, cell cycle stages, and relocalization at the nuclear lamina after mitosis [117]. 

As there is new evidence suggesting that pathogenic *LMNA* variants disrupt peripheral chromatin in specific cell types, further investigation of LADs could explain the largely varying phenotypes in laminopathies as well as the variable cell and tissue response to particular treatments (Figure 3C). Moreover, deregulation of LADs was recently reported in HGPS fibroblast cell lines, marking a newly recognized feature of epigenetic changes underlying the disease pathology [101] (Figure 3C). Interestingly, if LADs are a critical feature of the HGPS genome, they may hold a major missing link in connecting the previously established altered histone modifications, DNA methylation patterns, changes in gene regulation, and impact on disease-specific gene expression.

Kohler and colleagues were the first to report that epigenetic deregulation of LADs contributes to the molecular pathogenesis of HGPS and disease-specific gene expression [101]. This study used transposase-accessible chromatin (ATAC-see/-seq) to analyze chromatin accessibility and Infinium Methylation EPIC BeadChips to measure DNA methylation profiles in nine primary HGPS fibroblast cell lines with two parental and four age-matched control fibroblast cell lines. The results demonstrated both chromatin accessibility changes and alterations in DNA methylation enriched at the LADs (Figure 3C). Intriguingly, this study reported that DNA methylation alterations in HGPS were not randomly distributed, as they were primarily observed in regions that are lamin associated, partially methylated, and characterized by the presence of heterochromatic histone markers in dermal fibroblasts [101].

Investigation into DNA methylation and LADs was continued with the first genome-wide methylation analysis on peripheral blood DNA of eight classical HGPS patients and seven non-classical progeroid laminopathy patients with matched controls using Infinium Methylation EPIC arrays [118]. Initial methylation analysis comparing the eight classical HGPS and seven non-classical progeroid patients with their respective controls surprisingly revealed no significant differences in methylation patterns. However, a second aggregate analysis of the methylation sites of patients with progeroid laminopathies (classical and non-classical HGPS) revealed DNA methylation alterations at 61 CpG sites, which were associated with genes involved in the mTOR pathway. Interestingly, in contrast to Kohler and colleagues’ findings, this study reported no significant differences in DNA methylation patterns when comparing probes located in lamin A’s LADs and redistributed LAD genomic regions. However, Bejaoui and colleagues observed methylation differences at solo-WCGW CpG sites in partially methylated domains (PMDs) [118]. These are lamin-associated, late-replicating CpGs associated with methylation loss due to chronological aging and mitotic cell division in mammalian cells [115]. Moreover, the hypomethylation of solo-WCGW increases with age in almost all healthy cell types, serving as a universal marker to track the mitotic history of a cell [115]. In this HGPS patient’s erythrocyte DNA, the identified hypomethylated solo-WCGW CpG sites that were identified contradicted Kohler and colleagues’ reported findings of hypermethylation in solo-WCGWs CpGs of HGPS fibroblasts [101,118]. Taken together, these findings support previous reports of variability in LAD organization across different cell types, with an estimated 70% of LADs being constitutively organized, and the remaining facultative LADs exhibiting cell-type-specific genomic localization [119,120] (Figure 3C). This LAD dysfunction is also notable in non-HGPS states, such that LADs are redistributed in human cardiac myocytes from patients with dilated cardiomyopathies [121]. Moreover, these LADs were also marked by altered CpG methylation and differential gene expression. These findings are also significant for laminopathies, as DCM is a major cause of morbidity and mortality in these patients [121]. This also highlights the need for further analysis across multiple cell and tissue types to better understand differences in epigenetic dysregulation, which could perhaps explain phenotypic changes at the cellular, tissue, and organismal levels.

Taken together, these data suggest that a central factor in the molecular cause of HGPS could be the dysfunction at the LADs. However, the extent of its importance is yet to be experimentally determined. While epigenetic changes and gene alterations are being investigated, the connection of LADs with other shared cellular markers of progerin expression warrants further investigation. Therapeutic options have been identified to take advantage of 3D genome organization to improve the HGPS clinical phenotype.

## 6. Treatments

As the molecular mechanisms behind the pathogenesis of HGPS continue to be unraveled, several therapeutics have been developed in clinical and preclinical settings. The more effective and well-investigated therapeutics target different parts of the generation of progerin with the goal of eliminating it, as progerin exerts a dominant-negative effect on cellular phenotypes [47,122]. However, none of these therapies have been curative because of the failure to completely ameliorate the effect of progerin. Nevertheless, many of these therapies have been observed to be efficacious through improvement in clinical characteristics. This improvement is observed to be associated with progerin alteration/elimination, which is consistent with the corrections in chromosomal positioning and nuclear abnormalities. As mentioned previously, with respect to epigenetics, the importance of nuclear abnormalities in HGPS is of key significance, as the alterations of LADs result in gene-transcription changes [101]. The following sections describe the various therapeutics that alter progerin expression and/or processing, with a discussion on the relationship between molecular and clinical phenotype. The only drug that has been approved by the Food and Drug Administration (FDA) for the treatment of HGPS patients is lonafarnib, a farnesyltransferase inhibitor [123]. The urgency in the development of new therapies stems from the severe clinical manifestations of the disease along with the limited life expectancy.

### 6.1. Farnesyltransferase & GeranylGeranylTransferase Inhibitors

Farnesyltransferase inhibitors (FTIs) have primarily been used to treat cancers that develop from the aberrant function of the Ras protein, which needs a farnesyl group to be active [124]. The main mechanism is binding to the CaaX binding site of the farnesyltransferase (FTase), impeding the enzyme’s ability to interact with progerin [125] (Figure 2B). Thus, FTIs impede FTase’s ability to add a 15-carbon group to the cysteine residue of the CaaX box [25,126,127] (Figure 2B). In addition to cancers, they were proposed for HGPS therapy, as the farnesyl group will keep progerin permanently anchored to the nuclear lamina and thus induce various deleterious effects through interactions with lamin A/C, such as nuclear shape abnormalities [56,128]. These distortions can alter chromatin spatial organization at the LADs and thus induce genetic and epigenetic dysfunction [101]. Furthermore, FTIs have been shown to cause progerin and A-type lamins to re-distribute to the nucleoplasm [50,129]. 

FTIs’ efficacy was first noted in progerin-positive mouse embryonic fibroblasts and HGPS human fibroblasts cells, indicating a decrease in nuclear architectural abnormalities [50,63,130]. Additionally, FTIs were shown to improve chromosomal spatial positioning to the periphery in HGPS cells, although they are not effective for reducing DNA damage [49,131]. These observations are of importance, as farnesyltransferase inhibitors only ameliorate a component of progerin’s widespread cellular alterations. Furthermore, they have been shown to improve telomere mobility as well [91]. Despite these improvements, FTIs themselves cause deleterious effects, as they have been shown to cause the formation of binucleated cells with donut-shaped nuclei [130,131]. Binucleated cells are less inclined to proliferate, similar to HGPS cells [132]. Therefore, FTI’s deleterious effects could in fact act as a counterforce against its protective mechanisms. 

Further studies involved the creation of several mouse models to investigate the benefits of FTI treatment. When treated with FTIs, the ZMPSTE24-deficient and the *LMNA*^Hg/+^ models demonstrated maintenance of body weight and improvement in bone integrity, especially with rib fractures and grip strength [63,122]. Furthermore, vascular smooth muscle cell (VSMC) loss was prevented with FTI treatment when administered in young and 9-month-old mice harboring a transgene with the human lamin A G608G mutation [133]. These preclinical studies spurred the initiation of several human clinical trials. The first trial involved twenty-five children, at a mean age of seven, who received lonafarnib for a minimum of two years [134]. The results were encouraging, as about a third of the patients gained weight after administration of the drug. Increased cardiovascular health was also indicated, with improvements in peripheral arterial stiffness found through observations of pulse wave velocity. Patients also had improved skeletal rigidity and hearing [134]. Further analyses revealed an average lifespan increase of 1.6 years among lonafarnib-treated patients [95]. 

To further improve the treatment, several adjunctive therapies have been proposed to be utilized along with lonafarnib. An additional clinical trial was initiated to evaluate the efficacy of a triple therapy with lonafarnib, pravastatin, and zoledronate [135]. The addition of the latter two drugs is based on the mechanism that in the absence of farnesyltransferase, geranylgeranyltransferase prenylates progerin, which could still lead to the progeroid phenotype. This was further established by a reduction in nuclear defects of HGPS fibroblasts and improvement in the aged-associated phenotypes of ZMPSTE24-deficient mice treated with pravastatin and zoledronate [136]. Lamin A-G608G mutant mice that received triple therapy demonstrated improved bone and cartilage integrity parameters, in contrast to lonafarnib monotherapy [137]. Moreover, pravastatin alone causes a decrease in DNA damage markers in HGPS cells, meaning that it could complement FTIs to target nuclear defects [131]. The results of the clinical trial indicated an improvement in bone density when compared to monotherapy with lonafarnib; however, improvement in arterial stiffness of the carotid and femoral arteries was not as significant [135]. Importantly, a potential limitation of triple therapy is that an increased amount of the abnormal donut-shaped nuclei was observed when compared to FTI treatment alone [138].

Further combination therapies are currently being investigated. An ongoing clinical trial involves combination therapy of lonafarnib and everolimus [139]. The basis for this is that everolimus targets similar pathways as rapamycin, which has been shown to improve nuclear alterations, redistribute H3K27me3 levels, reduce DNA damage, and promote cellular growth [105]. Rapamycin induces the insoluble combination of lamin A/progerin to solubilize, resulting in its eventual clearance by autophagy. Moreover, FTIs’ deleterious effects of generating slowly proliferating binucleated cells as well as the inability to restore the nuclei’s response to mechanical stress are important targets of treatment [60,138]. Baricitinib has been proposed as a therapeutic agent along with lonafarnib, with significant improvements identified for DNA damage and cellular viability with combination therapy [140]. FTIs may potentially cause cardiotoxicity; however, this has not been further explored clinically [141]. Thus, farnesyltransferase inhibitors have been shown to be effective at improving symptoms and mortality in HGPS patients, and the focus now is on combination therapies with FTIs to identify further clinical improvement.

### 6.2. Isoprenylcysteine-Carboxyl-Methyltransferase Inhibitors

During the post-translational processing steps of prelamin A, isoprenylcysteine-carboxyl-methyltransferase (ICMT) adds a carboxy-methyl group to the terminal cysteine [142] (Figure 2B). Targeting inhibition of this step may ameliorate the disease phenotypes, as progerin remains not only permanently farnesylated but also carboxymethylated [143] (Figure 2B). HGPS fibroblasts with reduced ICMT activity or expression were observed to have increased proliferation of cells with progerin localization to the interior of the nucleus, interestingly without an improvement in nuclear morphology [144,145]. It is hypothesized that their protective mechanism derives mainly from improved AKT signaling, in contrast to rapamycin. This highlights the multiple mechanisms of the progerin molecule in initiating disease. Further studies were performed on ZMPSTE24-deficient mice and LMNA^G609G/G609G^ mice, both of which were homozygous for hypomorphic ICMT alleles (ICMT^hm/hm^). It was shown that these mice had improved body weight, grip strength, VSMC nuclei, muscle cross-sectional diameter, and survival, in contrast to mice with functioning ICMT alleles [144,145]. Treatment of the LMNA^G609G/G609G^ mice with an ICMT inhibitor (UCM-13207) reinforced similar findings as the previous HGPS mouse models, with the survival rates both being 100% at 140 days [146]. This demonstrates the potency of this chemical inhibitor in ameliorating the HGPS phenotype as it can be as effective as the genetic-based models. In addition, the study showed a decrease in progerin expression in multiple tissues, including the aortic arch [146]. ICMT inhibitors have been revealed to be multifaceted in treating the phenotypes of HGPS; however, their efficacy derives from other mechanisms besides altering the nuclear architecture. Furthermore, ICMT inhibitors have only been tested in mouse models and it is unknown how the clinical phenotypes will be altered in HGPS patients.

### 6.3. Antisense Oligonucleotides 

Improper splicing is the main mechanism that results in the generation of progerin (Figure 2B). Morphilino antisense oligonucleotides (ASO) attempt to resolve this by base-pairing with the mRNA transcript of progerin to alter splicing dynamics [147] (Figure 2B). For preclinical HGPS cell lines, ASOs were initially developed to target exon 11 of *LMNA*, as lamin A is dispensable. The cell lines were observed to have reduced prelamin A and progerin with increased lamin C expression [128]. Furthermore, improvements were observed in nuclear shape, levels of lamin A-associated proteins, and heterochromatin markers [55,128]. Most interestingly, proper expression was restored for several genes (e.g., MMP14, MMP3, CCL8, and HASIII) that are involved in connective tissue homeostasis [55]. The restoration of the function indicates that improvement of the spatial localization of the genes leads to the cells being re-established with clinical symptomatic improvement. Additionally, a study indicated that combination therapy of ASOs targeting the exon 10 cryptic splice donor site and the cryptic splice site at exon 11 showed a significant improvement in nuclear abnormality, along with monotherapies of those ASOs [148]. Furthermore, ASOs were tested on the cells of patients with HGPS-like manifestations with varying mutations on the *LMNA* gene [149]. Examples include transcripts of prelamin AΔ35 and prelamin AΔ90. Again, improvements in nuclear morphology were noted along with a reduction in altered prelamin A [149]. Recently, a modified ASO was developed, termed peptide-conjugated phosphorodiamidate morpholino oligomers (PPMOs) [150]. Treatment of one PPMO (SRP-2001) illustrated a loss in progerin transcript expression by 92% in human HGPS fibroblasts as well as improvements in cellular growth and lamin B expression [150]. In vivo studies with LMNA^G609G/G609G^ mice demonstrated that non-PPMO ASOs improved lifespan, body weight, and appearance along with a decrease in prelamin A expression [128,148]. In one study, ASO delivery was effective in the heart, kidney, and liver, by measurement of progerin expression, in stark contrast to CRISPR-Cas9 and ABE treatments (discussed in the next section); however, PPMO delivery was not very effective to the kidney and heart, but was excellent to the aorta [148,150]. This may be due to structural differences between these two types of ASOs [148,150]. Furthermore, PPMO treatment on transgenic G608G *LMNA* mice demonstrated similar results, as well as improvements in VSMC density and adventitial fibrosis [150]. Recently, a library was generated of potential ASOs to determine which are most effective, based on alterations in lamin A, progerin, and lamin C mRNA levels [151]. Interestingly, the most potent ASO (L-B143) was determined to target a sequence that is part of both intron 11 and exon 12, unlike the ones utilized in previous studies. The efficacy of L-B143 was tested with G608G *LMNA* with a reduction in progerin mRNA levels observed. However, its effects on protein levels in different organs was variable. Furthermore, L-B143 showed sex-specific improvements in lifespan, more so in males than females. However, it was ineffective in abrogating the vascular manifestations of the disease [151]. Overall, ASOs have excellent potential as a therapeutic, but require further investigation, as they have only been tested in preclinical models and target different sites on the LMNA mRNA, resulting in various manifestations. Therefore, a combination of ASOs would be the most appropriate therapy.

### 6.4. CRISPR-Cas9 and Adenine Base Editors 

In HGPS, the point mutation (1824C>T) in the *LMNA* gene leads to the creation of progerin (Figure 2B) [46,142]. Progerin behaves in a dominant-negative manner in the pathogenesis of HGPS [47,55]. Hence, it is important to target the *LMNA* gene and its initial products to prevent the expression of progerin. Two strategies have been developed and tested in mice/human cells and mouse models for this purpose: CRISPR/Cas9 and adenine base editors (ABEs). 

Two groups independently developed CRISPR-Cas9 strategies [152,153]. One group used a guide RNA (gRNA) targeting exon 11, while the other used two gRNAs, selecting for either exon 11 or 12 [152,153]. This strategy is based on the fact that lamin A is dispensable for normal cellular function [122]. Preclinical studies showed that this strategy reduced progerin levels with concurrent amelioration of nuclear defects in murine embryonic and HGPS human fibroblasts [153]. Furthermore, in vivo delivery of the CRISPR-Cas9 components was achieved by packaging it in an adenovirus-associated virus-9 vector (AAV9) with LMNA^G609G/G609G^ mice [152,153]. Both observed differences in tropism of the vector for different organs, with the one demonstrating high tropism for the liver, heart, and muscle but low tropism for the aorta and lung [152,153]. This is a potential limitation for this therapy, as vascular pathology is the most significant risk factor for mortality in these patients. The mice demonstrated improvements in survivability, weight gain, phenotypic appearance, and muscle grip, compared to age-matched non-treated controls [152,153]. Furthermore, Beyret and colleagues demonstrated that the degeneration of vascular cells was lessened with the CRISPR-Cas9 therapeutic, which could reflect the benefits of using multiple gRNAs [152]. These findings demonstrated the possibility of utilizing the CRISPR-Cas9 genomic editing tool to reduce symptoms and improve mortality in HGPS patients. The efficacy of this treatment is not superior to the previous studies in terms of weight gain and life expectancy with mouse models. 

Correcting the actual mutation through gene editing is another possibility to abrogate progerin expression. The superiority of this technique to CRISPR-Cas9 is that it does not induce the negative consequences of frameshift mutations. One group developed helper-dependent adenoviral vectors (HDAdVs) that utilize homologous recombination to reverse mutations in the *LMNA* gene [154]. This was tested on iPSC-derived vascular smooth-muscle cells, demonstrating an elimination of progerin as well as a reduction of senescent cells and nuclear shape alterations [154]. Furthermore, a novel approach to prevent progerin expression entails the use of adenine base editors (ABE) [155]. Adenine base editors convert A-T to a C-G sequence with the use of a tRNA adenosine deaminase, sgRNA, and an altered-Cas9 molecule. Patient-derived HGPS fibroblasts treated with an ABE exhibited decreased progerin expression and fewer defective nuclei. In vivo therapy was administered via an AAV9 lentiviral vector to P3 and P14 mice. Thus, much like the CRISPR-Cas9 tools, the ABE showed more tropism for the liver and heart but less so for the aorta. Regarding the phenotypes, the treated P14 mice interestingly showed an improvement in maintenance in VSMCs and adventitial fibrosis that mirrored the wild-type. Additionally, lifespan doubled for the P14-treated mice. P3 mice also reflected improvements with VSMC maintenance and lifespan, but less so than P14 [155]. Another study extended the effectiveness of ABEs in correcting the mutation in keratinocytes and B lymphocytes [156]. Improvement of progerin was noted with treatment, but this did not extend to escalating doses. This group aimed to correct the dermatological phenotypes of transgenic mice that only express progerin in keratinocytes. The treated mice demonstrated improvements in epidermal thickness and inflammation. Accordingly, there was a decrease in the amount of progerin transcript in the skin of treated mice, but not the protein itself. Additionally, treated mice with progerin-free keratinocytes at certain levels of the skin revealed decreased DNA damage and increased expression of keratin 15 [156]. Therefore, ABEs represent an innovative strategy for treating the origin of the disease, with minimal risk for causing further alterations to the genome. Further studies need to elucidate the efficacy of treatment, as it looks promising, especially in terms of lifespan.

The therapeutics discussed above, which target progerin production, have shown to be beneficial in ameliorating specific clinical characteristics and improving survival and body weight. However, there are distinctions noted. It is difficult to directly compare whether or not a specific therapeutic is superior to one another, as each study used different methods. Nevertheless, the observation that ABEs can more than double the lifespan of transgenic HGPS mice to match the elderly age of wild-type mice, along with their lack of off-target effects, give credence to its ability to be the most effective [155]. ASOs and CRISPR-Cas9 were the only therapeutics where life expectancy was studied, with the extension being maximized at about ~60 (ASOs) and ~50 (CRISPR/Cas9) percent, respectively [150,152]. CRISPR-Cas9 does not match the ABEs due to the development of an enlarged GI system, causing the mice to die suddenly due to the decreased tropism of the gRNAs in the colon [152]. Regarding other clinical parameters of HGPS, ABEs were only studied to reverse aortic pathology and maintain body weight, mimicking the wild-type phenotype. 

Each of these individual drugs has its own limitations, some of which are intrinsic and can be difficult to address. However, the CRISPR-Cas9 strategies and ABEs demonstrated diminished tropism for various organs. The exact mechanism for this is unknown, but it may stem from the use of the AAV9 vector for delivery. This vector is known to be able to infect many tissues and organs in the body, but AAV9 has been identified to have increased tropism for the liver and induce an immunological response, which may prevent infection of target tissue [157,158]. The importance of developing a vehicle to effectively deliver these therapeutics cannot be understated. 

### 6.5. Future Perspectives of Therapeutics 

While many of these therapies have been shown to be efficacious in ameliorating the HGPS phenotypes, they are not a cure. In the near future, the solution to fully reversing the clinical manifestations will likely include combinations of multiple therapies. Numerous FTI combination therapies have already been tested with mixed results; thus, further testing with all types of HGPS therapeutics is warranted. These might include ICMT inhibitors with FTIs, as ICMTs were shown not to ameliorate the nuclear defects, while FTIs were not efficacious at reducing DNA damage. In combination, both therapeutics would eliminate these two important factors in the clinical pathogenesis of HGPS. Combination can extend to gene-based therapies as well, as they were not completely effective in abrogating progerin expression, and FTIs can act as a backup to prevent progerin’s deleterious effects. Several other therapies can also be utilized for combination. For example, tocilizumab inhibits interleukin-6, a cytokine up-regulated in aged individuals, improving both nuclear deformities and defective DDR [159]. Furthermore, it showed impressive improvements in cardiovascular and skeletal pathology in mice [159]. Additionally, progenin, a drug that blocks the interaction of lamin A and progerin, demonstrates an improvement in homozygous progeroid mice longevity by 10 weeks [160]. Furthermore, inhibitors such as MnBAP (superoxide dismutase mimetic) can target ROS generation and mitochondrial dysfunction induced by progerin. Moreover, MnBAP supplemented baricitinib by reducing nuclear defects [161]. Gene-based therapies can serve adjunctively as well. hTERT therapy was tested on vascular endothelial cells (ECs), showing improvements in global gene expression, morphology, growth, and DDR, as well as cell-specific markers such as the creation of nitric oxide [162]. Improvements were also noted for a mouse model [162]. Furthermore, up-regulation of SIRT7 in vascular ECs showed improvements in inflammation and extension of lifespan by 76% [163]. Overall, each HGPS patient may be more responsive to one treatment compared to another. Therefore, a curative treatment will likely consist of a drug cocktail that is individualized for each patient. 

## 7. Conclusions

HGPS only mimics normal aging in the connective tissue components of the body [10,11]. However, in those specific tissues, progeria serves as an excellent model for the study of aging. Accordingly, the molecular alterations (i.e., the hallmarks of aging) between the two conditions are partially shared, as well as many of the mechanisms by which they are derived [1]. Furthermore, progerin itself has been identified in normally aged fibroblasts [56]. However, there are other mechanisms to how normal aging manifests, and progeria cannot be seen as an exact replica. Nevertheless, a better understanding of the epigenetic landscape of progeria is crucial, as its intimate relationship with other changes at the cellular level has yet to be explored and could further our understanding of the complex nature of aging. The various therapeutics developed to inactivate or abrogate progerin have been shown to ameliorate many of the defective cellular phenotypes. Clinically, these interventions have had promising results, although current therapeutics fall short of an actual cure. A better understanding of the underlying molecular and cellular mechanisms is still needed, as additional pathways may be targeted. Only then will the most efficacious treatment for HGPS patients be actualized and, in the near future, potentially be prescribed to normal-aging individuals. 

## Data Availability

Not applicable.
